# Urantide alleviates lipopolysaccharide/D-galactosamine-induced acute liver failure through upregulating carboxylesterase1f in mice

**DOI:** 10.3389/fcimb.2025.1653725

**Published:** 2025-12-17

**Authors:** Wanhua Yang, Shu Bian, Liangming Liu

**Affiliations:** Songjiang Hospital Affiliated to Shanghai Jiaotong University School of Medicine, Shanghai, China

**Keywords:** acute liver failure, urotensin II, UII receptor, Ces1f, mouse

## Abstract

**Aims:**

To investigate the effect of urotensin II(UII)/UII receptor(UT) on hepatic carboxylesterase1f(Ces1f) expression in acute liver failure(ALF) mice.

**Methods:**

ALF was induced in male Balb/c mice using lipopolysaccharide (LPS)/D-galactosamine (D-GalN) i.p. after a tail vein injection of Urantide, a specific antagonist of UT receptor. Liver tissues were collected at 0-12h to detect *UII* and *Ces1f* mRNA levels in ALF mice. Mice were divided into four groups (n=6 each group): (A) Urantide(-), LPS/D-GalN(-), (B) Urantide(+), LPS/D-GalN(-), (C) Urantide(-), LPS/D-GalN(+), and (D) Urantide(+), LPS/D-GalN(+). Liver tissues were collected at 6 h after a challenge of LPS/D-GalN. Time-depended levels were observed of hepatic *UII* and *Ces1f* mRNA over a period of 12 hours after LPS/D-GalN challenge. Liver injury was evaluated via hematoxylin-eosin (HE) staining, serum alanine aminotransferase(ALT)/aspartate aminotransferase(AST), and the mRNA and protein expression levels of Ces1f and UII expression were determined by fluorescence *in situ* hybridization(FISH), quantitative real-time PCR (qPCR) and Western blotting (WB), respectively.

**Results:**

After LPS/D-GalN injection, hepatic *UII* mRNA rose at 2 h (*P* < 0.05 *vs* 0 h), reached the peaked level at 6 h, and the level began to degrade at 8 h, but remained higher than at 0 h (*P* < 0.05, 10 h *vs* 0 h) till 12 h (*P*>0.05, 12 h *vs* 0 h); while hepatic *Ces1f* mRNA decreased at 6 h (*P* < 0.05 *vs* 0 h), reached the lowest level at 10 h, but began to rise at 12 h (*P*>0.05, 12 h *vs* 10 h). In addition, urantide pretreatment inhibited the up-regulated expressions of hepatic *UII* mRNA and protein, whereas increases the down-regulated expressions of hepatic *Ces1f* mRNA and protein induced by LPS/D-GalN attack at 6 h.And serum ALT and AST levels were significantly decreased, whereas hepatic inflammatory injury improved via urantide injection in LPS/D-GalN-induced ALF mice.

**Conclusion:**

Ces1f maybe negatively regulated by UII/UT signal in LPS/D-GalN-induced ALF.

## 1 Introduction

Acute liver failure (ALF) is an acute onset condition characterized by severe liver damage caused by multiple factors, leading to significant impairment or decompensation of the liver’s synthetic, detoxification, metabolic, and biotransformation functions. As the body’s largest immune organ, hepatic damage involves immune-mediated inflammatory mechanisms exerted by hepatic non-parenchymal on parenchymal cells underlying the onset and progression of ALF (Wu et al., 2010). In the liver, Kupffer cells (KC), the liver’s resident macrophages, are the most important non-parenchymal cells, playing a pivotal role in the innate immue. An innate activation is the most early event triggering hepatic inflammatory injury (Triantafyllou et al., 2018a, Triantafyllou et al., 2018b; Antoniades et al., 2014). Under the stimulation of liver injury factors (including lipopolysaccharide (LPS)), KCs are activated and undergo M1 polarization (inflammatory phenotype), releasing large amounts of pro-inflammatory cytokines (such as tumor necrosis factor α(TNF-α), interleukin 1β (IL-1β), interferon γ (IFN-γ), interleukin 6 (IL-6), etc.) and chemokines (e.g.,C-X-C motif chemokine ligand 1 (Cxcl1), C-C motif chemokine ligand (Ccl3), C-C motif chemokine receptor-like 2 (Ccrl2), etc.), working in concert with acquired immune cells to eliminate common pathogen-associated molecular patterns (PAMPs) and damage-associated molecular patterns (DAMPs) in the body (Parlar et al., 2023; Li et al., 2023).

In ALF mice, KCs express Urotensin II (UII) at high levels. UII is a cyclic polypeptide molecule containing 11 amino acids with neurohormone-like biological activity.It plays a significant role in various pathophysiological activities, such as regulating immune function and participating in the regulation of hypertension and renal fibrosis (Sun and Liu, 2019). The UII receptor (Urotensin receptor, UT) is a specific orphan G protein-coupled receptor that binds specifically to UII to mediate signal transduction. Cells expressing UII and UT include endothelial cells, hepatobiliary epithelial cells, and KCs in the liver (Sun and Liu, 2019). Research confirms that UII primarily mediates hepatic immune-inflammatory injury by activating the toll-like receptor 4 (TLR4) signaling pathway and releasing its downstream proinflammatory cytokines, including TNFα and IL-1β (Liang et al., 2013). The hepaticellular distribution patterns of UII and UT show remarkable consistency, suggesting potential regulation by autocrine and paracrine mechanisms. As the liver’s innate immune cells, KCs have been identified as the primary cellular source of UII/UT and inflammatory cytokine expression. Urantide, a specific antagonist of UT, can effectively inhibit the UII/UT signal and the release of these inflammatory mediators (Yu et al., 2020).

During ALF, hepatic metabolism is significantly impaired, including drug metabolism and lipid metabolism. Carboxylesterase 1 (Ces1), as a primary metabolic enzyme in the liver, is synthesized and highly expressed in the liver (Collins et al., 2022). It promotes the metabolism of various environmental toxins, carcinogens, and drugs (e.g., clopidogrel, sacubitril/valsartan, oseltamivir) (Liu et al., 2024) and participates in the transport and metabolism of cholesterol esters and free fatty acids (Satoh et al., 2002; Sanghani et al., 2009; Satoh and Hosokawa, 1998). Ces1f, a subtype of carboxylesterase 1, exhibits hydrolytic activity toward triglycerides and retinol (RE) (He et al., 2019; Crow et al., 2010; Mangum et al., 2018). Study indicate Ces1f plays an important role in liver X receptor α(LXRα) protection of hepatic damage (Becares et al., 2019). Our reporter showed that KCs expressed Ces1f, and hepatic damage aggravated due to *Ces1f* gene knockdown in the liver. It suggests Ces1f has hepatic protection against immune infammation via KC activation. However, the effect of UII/UT on Ces1f remains unclear.

## This study aims to investigate *Ces1f* gene expression in LPS/D-GalN-induced ALF mice model using UT antagonist urantide, thereby further elucidating the pathophysiological mechanisms through which the UII/UT system mediates ALF development.

## 2 Materials and methods

### 2.1 Reagents

Lipopolysaccharide (LPS) and D-galactosamine (D-GalN) were purchased from Sigma-Aldrich (St. Louis, MO,USA). Urantide was obtained from Peptides International (Tokyo, Japan). The SYBR Green PCR Master Mix and reverse transcription kit were acquired from TaKaRa Bio Inc. (Tokyo, Japan). Trizol reagent was procured from Thermo Fisher Scientific (Shanghai, China). All PCR primers were commercially synthesized by Shanghai Bioengineering Co. Ltd.(Shanghai, China).

### 2.2 Animals experiments

Healthy male Balb/c mice, aged 6–8 weeks, specific pathogen-free (SPF) grade, weighing 20–22 g, were obtained from the Experimental Animal Center of Shanghai First People’s Hospital Affiliated to Shanghai Jiao Tong University (License No.SCXK (Hu) 2018-0004). The mice were housed under controlled environmental conditions: temperature (20–22 °C), noise (<50 dB), humidity (40–80%), and a 12/12-hour light/dark cycle. They were provided with ad libitum access to food and water. Before the experiment, the mice were fasted for 12 hours with free access to water. Animal experimental protocols were approved by the Animal Welfare and Ethics Committee of Songjiang Hospital Affiliated to Shanghai Jiao Tong University School of Medicine (Approval No. ACE-001-2024-R1).

A total of 42 healthy male Balb/c mice were intraperitoneally administered LPS (50 μg/kg) combined with D-GalN (800 mg/kg). Liver tissue samples were collected at specified time points (0, 2, 4, 6, 8, 10, and 12 h; n=6 per time point) for analysis of *UII* mRNA and *Ces1f* mRNA expression levels.An additional cohort of 24 healthy male Balb/c mice were randomly allocated into four experimental groups (n=6 per group): Group A: urantide(-) LPS/D-GalN(-); Group B: urantide(+) LPS/D-GalN(-); Group C: urantide(-) LPS/D-GalN(+); Group D: urantide(+) LPS/D-GalN(+). The ALF mice model was established according to established protocols (Sanghan et al.,2009) through intraperitoneal injection of LPS (50 μg/kg) and D-GalN (800 mg/kg) dissolved in 0.2 mL of 0.9% sodium chloride solution. Control animals received an equivalent volume (0.2 mL) of 0.9% sodium chloride solution alone. For pretreatment groups, urantide (0.6 mg/kg dissolved in 0.9% sodium chloride) was administered via tail vein injection 30 minutes prior to LPS/D-GalN or saline administration. Liver tissue specimens and serum samples were collected 6 hours post-treatment.

### 2.3 Biochemical assays

Frozen serum samples from each experimental group were retrieved from -80 °C storage and thawed at room temperature. The serum was then diluted to an appropriate volume with 1× phosphate-buffered saline (PBS). Subsequently, the samples were sent to the Clinical Laboratory of Songjiang District Central Hospital (Shanghai, China) for automated biochemical analysis. Serum alanine aminotransferase (ALT) and aspartate aminotransferase (AST) levels were measured using a fully automated biochemical analyzer. The obtained values were multiplied by the corresponding dilution factor to determine the actual serum ALT and AST concentrations.

### 2.4 Patholigical examination

The tissue microarray sections were sequentially immersed in xylene and graded ethanol solutions for deparaffinization and rehydration. Following antigen retrieval, immunohistochemical staining was performed using 3,3’-diaminobenzidine (DAB) as the chromogen with hematoxylin counterstaining. The stained sections were then dehydrated through an ethanol gradient, cleared in xylene, and mounted with neutral balsam under cover slips. All histological assessments were conducted in a blinded manner by certified pathologists from our institution, who randomly selected 8–10 high-power fields (×400 magnification) for evaluation under light microscopy.

### 2.5 Reverse transcription quantitative polymerase chain reaction analysis

For RNA isolation, 50 mg of liver tissue was homogenized in 1 mL of Trizol reagent (Takara R036A) following the manufacturer’s protocol, with all procedures performed on ice to prevent RNA degradation. The extracted total RNA was resuspended in 50 μL of RNase-free water, and its concentration and purity were spectrophotometrically determined. All samples exhibited A260/A280 ratios >1.8, indicating high RNA purity. First-strand cDNA synthesis was performed using 500 ng of total RNA with 2 μL of 5× PrimeScript RT Master Mix in a 10 μL reaction volume (adjusted with DEPC-treated water). The reverse transcription conditions were: 37 °C for 90 s, 85 °C for 5 s, followed by indefinite hold at 4 °C. The resulting cDNA was stored at -20 °C until use.Gene-specific primers (designed and synthesized by ShanghaiBioengineering Co.) with their corresponding sequences and product lengths are listed in **Table 1**. Quantitative real-time PCR was performed using the SYBR Green system(Takara RR420) in a laminar flow hood with reagents maintained on ice. The 20 μL reaction mixture contained:10 μL of TB Premix Ex Taq (2×),0.4 μL each of forward and reverse primers,0.4 μL of ROX Reference Dye II (50×),2 μL of cDNA template,RNase-free water to final volume.Amplification was conducted on an ABI7500 instrument using a two-step protocol:Initial denaturation: 95°C for 15 min (1 cycle);Amplification: 40 cycles of 94°C for 20 s and 60°C for 34 s.*GAPDH* served as the endogenous reference gene. Following amplification, CT values were obtained and relative mRNA expression levels were calculated using the 2^-ΔΔCT^ method.

**Table 1:** Primer sequences used for real-time PCR analysis.

Gene	Primer sequence (5’-3’)	Product (bp)
*Ces1f*	Forward: TGGAGAGTCAGCAGGAGGTTACAG	290
	Reverse: AGCACACTTGTCACGAACTGGTC	
*UII*	Forward: CTTCTCGCCGCATCATGGACAG	168
	Reverse: TGACGGGAAGGGACAGCAGTG	
*GAPDH*	Forward: GACATGCCGCCTGGAGAAAC	244
	Reverse: GTCCACCACCCTGTTGCTGTAG	

### 2.6 Fluorescence *in situ* hybridization

Fresh liver tissues were immersed in fixative solution for ≥24 h at 4°C,then dehydrated through a graded ethanol series (70% to 100%),cleared in xylene, and infiltrated with paraffin (58–60°C) using an automated tissue processor. Tissues were embedded in paraffin blocks. Briefly, molten paraffin was poured into molds, and tissues were oriented before cooling on a -20°C freezing stage. Solidified blocks were trimmed for sectioning. Serial sections (5μm thickness) were dried at 60°C for 45 min and then dewaxed in xylene (2×10 min) and rehydrated through a graded ethanol series (100% to 70%).Slides were treated with 3% hydrogen peroxide (H_2_O_2_) in methanol for 10 min at room temperature (RT) to block peroxidase activity. Target mRNA was exposed by incubating sections in citrate buffer (pH 6.0) at 95°C for 15 min. Sections were covered with prehybridization buffer and incubated at 37°C for 4 h in a humidified chamber to reduce nonspecific binding. Digoxigenin (DIG)-labeled Ces1f probes (100 ng/μL in hybridization buffer) were applied, and slides were hybridized at 60°C overnight (16–18 h).Stringency washes: 2× SSC (RT, 10 min) → 0.5× SSC (60°C, 15 min).Blocking: 5% bovine serum albumin (BSA) in PBS for 1 h at RT. Probes were detected with biotinylated anti-DIG antibody (1:500, Roche) for 1 h at RT. Nuclei were counterstained with DAPI (5 μg/mL, 2 min).Slides were cover slipped with antifade mounting medium (Thermo Fisher) and imaged under a Zeiss LSM 880 confocal microscope (20×/40×objectives).

### 2.7 Western blotting

Approximately 20 mg of liver tissue was placed in a 1.5 mL tube. Added 1 mL of RIPA buffer supplemented with 50 μL of protease inhibitor cocktail (Roche). Tissue was mechanically disrupted using three 3-mm stainless steel beads in a tissue homogenizer (60 Hz, 2 min), followed by incubation on ice for 30 min to complete lysis. Lysates were centrifuged at 12,000 × g, 4°C for 10 min. Supernatants were collected and stored at −80°C until use. Protein concentration was determined using a BCA assay, following the manufacturer’s protocol. Samples were mixed with 4× Loading buffer and heated at 95°C for 5 min. Equal amounts of protein (20–30 μg/lane) were resolved on 12% polyacrylamide gels at 100 V for 90 min. Proteins were electrophoretically transferred to nitrocellulose membranes. Membranes were incubated in 5% non-fat milk in TBST for 1 h at RT. Rabbit monoclonal anti-Ces1f (1:1000, Abmart) and Rabbit monoclonal anti-UII (1:1000, Santa Cruz)Diluted in 5% BSA/TBST; incubated overnight at 4 °C with gentle agitation. HRP-conjugated goat anti-rabbit IgG (1:2000, beyotime) in 5% milk/TBST, 1 h at RT. Membranes were incubated with SuperSignal™ West Pico PLUS Chemiluminescent Substrate (Thermo Fisher) for 1 min and imaged using a Bio-Rad ChemiDoc™ MP System. Antibodies were removed with stripping buffer at RT for 10 min. Reblocked and reprobed for β-actin (1:10000, beyotime) or GAPDH (1:10000, beyotime) as loading controls. Band intensities were quantified using ImageJ 1.4.3.67 (NIH).

### 2.8 Statistical analysis

Quantitative data were expressed as mean ± standard deviation (SD) and analyzed using SPSS 20.0 software (IBM Corp., USA). For comparisons between two groups, independent samples t-test was used for parametric data, while the Mann-Whitney U test was applied for non-parametric data. Multiple group comparisons were performed using one-way ANOVA followed by appropriate *post hoc* tests (Tukey’s or LSD) when significance was detected; when the assumption of homogeneity of variance was violated (as assessed by Levene’s test), the Kruskal-Wallis test was employed instead. The P-value < 0.05 was considered statistically significant for all analyses.

## 3 Results

### 3.1 Time-dependent expression of hepatic *UII* mRNA in LPS/D-GalN-induced ALF mice

**Figure 1:**
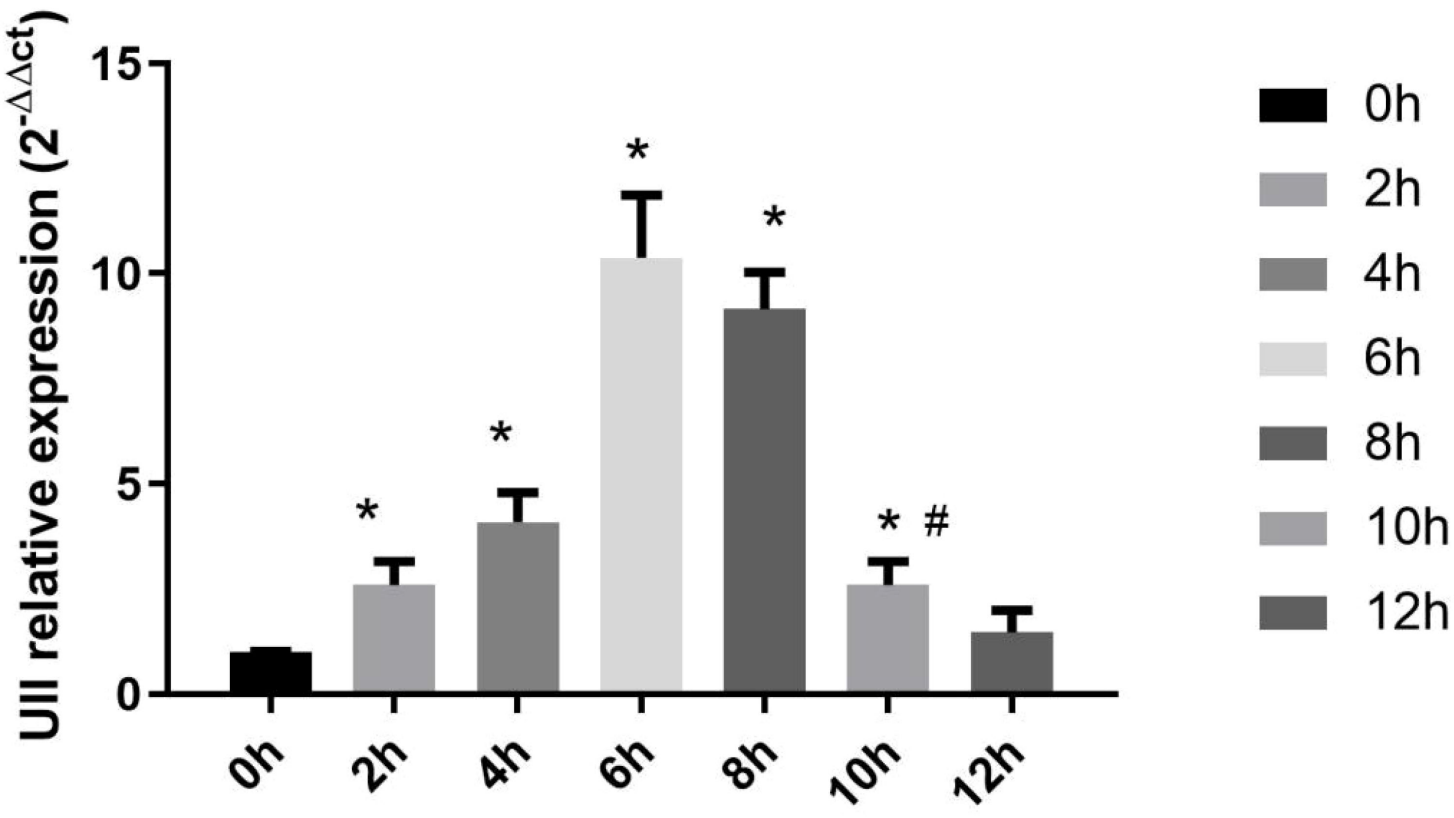
Hepatic *UII* mRNA Expression in LPS/D-GalN-Induced ALF Mice at Different Time Points. The vertical axis represents the relative expression level of *UII* 2^-△△ct^, *Compared to 0 h *P*<0.05, #Compared to 8 h *P*<0.05.

Following LPS/D-GalN administration, hepatic *UII* mRNA expression exhibited a time-dependent dynamic pattern, with relative expression levels of 1 (baseline), 2.62 ± 0.61, 4.09 ± 0.71, 10.38 ± 1.49, 9.15 ± 0.88, 2.56 ± 0.57, and 1.46 ± 0.52 at 0, 2, 4, 6, 8, 10, and 12 h post-treatment, respectively. Statistical analysis revealed a significant upregulation as early as 2 h (*P* < 0.01 *vs* 0 h), peaking at 6 h (10.38 ± 1.49, *P* < 0.01) and remaining elevated at 8 h. Expression levels subsequently declined, showing significant reduction at 10 h (*P* < 0.05 *vs* 0 h) and returning to baseline by 12 h (*P* = 0.435 *vs* 0 h) (**Figure 1**). This biphasic response suggests rapid activation and subsequent resolution of *UII* signaling following inflammatory challenge.

### 3.2 Time-dependent expression of hepatic *Ces1f* mRNA in LPS/D-GalN-induced ALF mice

**Figure 2:**
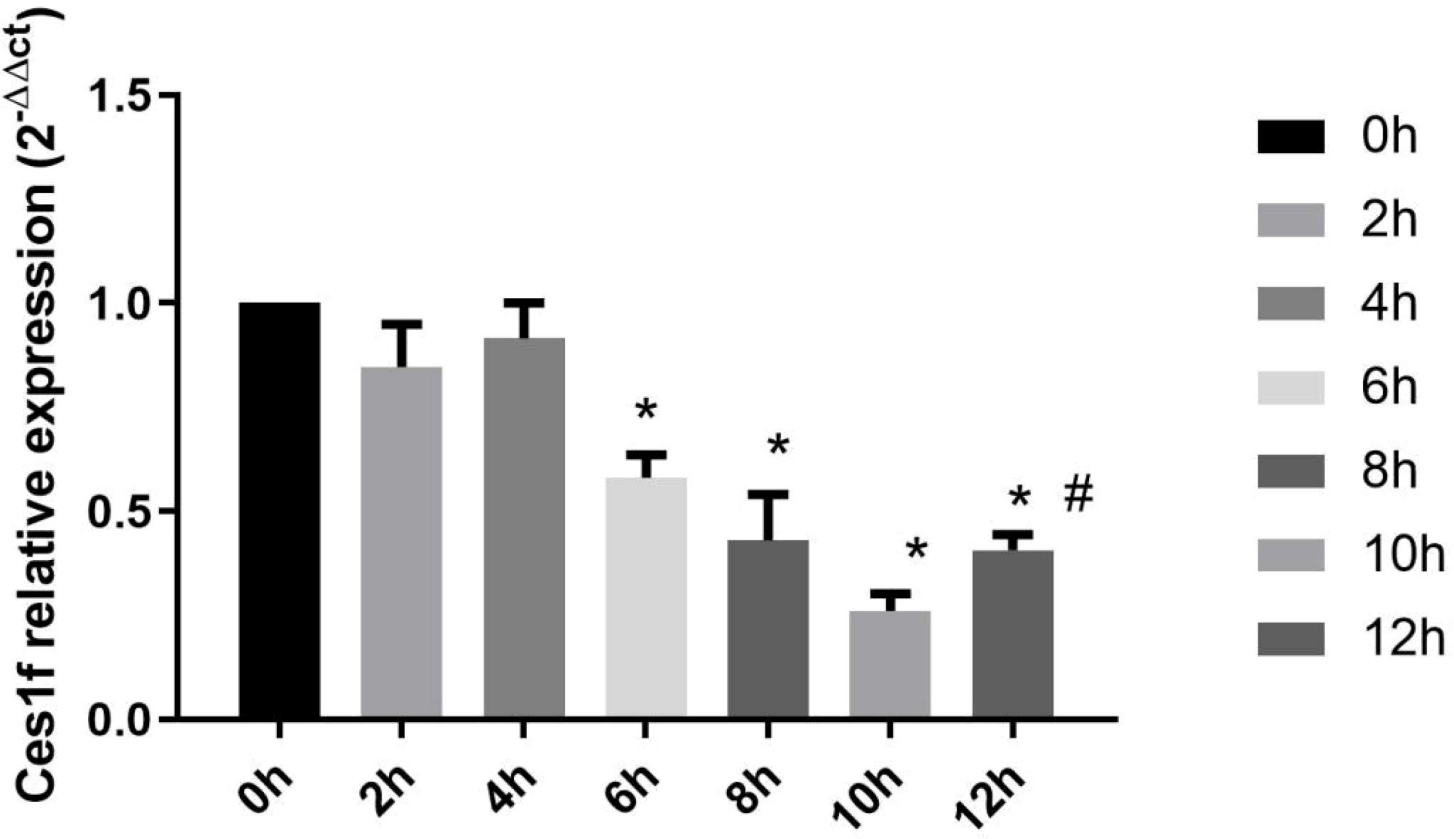
Hepatic *Ces1f* mRNA Expression in LPS/D-GalN-Induced ALF Mice at Different Time Points. The vertical axis represents the relative expression level of *Ces1f*^2-△△ct^, *P < 0.05 *vs* 0 h, #*P* < 0.05 *vs* 10 h.

Following LPS/D-GalN challenge, hepatic *Ces1f* demonstrated a progressive downregulation, with relative expression levels measuring 1 (baseline), 0.85 ± 0.10, 0.92 ± 0.09, 0.58 ± 0.05, 0.43 ± 0.11, 0.26 ± 0.04, and 0.41 ± 0.04 at 0, 2, 4, 6, 8, 10, and 12 h post treatment, respectively. Statistical analysis revealed this suppression became statistically significant by 6 h (*P* < 0.01 *vs* 0 h), reaching a nadir at 10 h (0.26 ± 0.04), remained during the observed time window (all *P* < 0.05 *vs* baseline). Notably, the expression levels began to rise at 12 h (0.41 ± 0.04, *P* < 0.05 *vs* 10 h). These findings demonstrate that *Ces1f* downregulation (1) initiates till 6 h, (2) correlates temporally with ALF progression, and (3) exhibits a statistically reversion at the end of the observed timeframe (**Figure 2**), suggesting its expression kinetics may serve as an indicator of hepatic metabolic dysfunction or recovery during inflammatory injury.

### 3.3 Hepatic *UII* mRNA expression in mice challenged by LPS/D-GalN via urantide pretreatment

**Figure 3:**
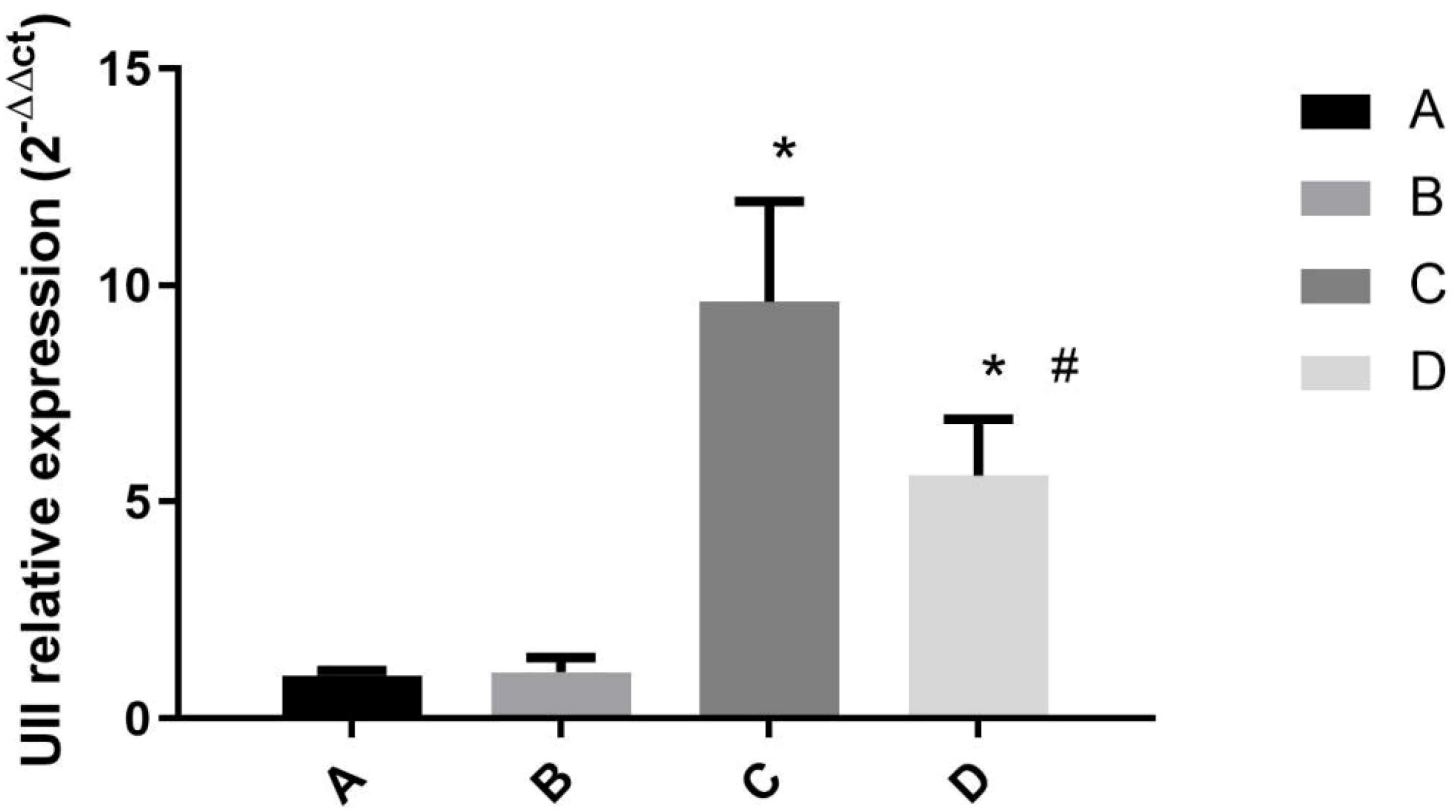
Expression of *UII* mRNA in liver tissue of mice treated with LPS/D-GalN at 6h. **(A)** Urantide(-)LPS/D-GalN(-); **(B)** Urantide(+)LPS/D-GalN(-); **(C)** Urantide(-)LPS/D-GalN(+); **(D)** Urantide(+)LPS/D-GalN(+); *P < 0.01 *vs* group A; #*P* < 0.05 *vs* group C;The vertical axis represents the relative expression levels of *UII* 2^-△△ct^.

Quantitative analysis of hepatic *UII* mRNA expression revealed significant differences among experimental groups, with relative expression levels of 1.00 (Group A), 1.08 ± 0.33 (Group B), 9.62 ± 2.31 (Group C) and 5.61 ± 1.3 (Group D). Statistical evaluation demonstrated that: (i) LPS/D-GalN-challenged Group C exhibited a 9.6-fold upregulation compared to controls (*P* < 0.01 *vs* Groups A/B); (ii) no significant difference existed between untreated controls (Group A *vs* B, *P* = 0.99); and (iii) urantide pretreatment in Group D significantly attenuated this drug induction by 41.7% versus Group C (*P* < 0.05) (**Figure 3**). These results establish that: (1) UII is robustly upregulated during ALF, (2) basal UII expression remains stable under physiological conditions, and (3) pharmacological UT receptor blockade partially reverses inflammation-induced UII overexpression, supporting the involvement of UII/UT signaling in ALF pathogenesis.

### 3.4 Hepatic *Ces1f* mRNA expression in mice challenged by LPS/D-GalN via urantide pretreatment

**Figure 4:**
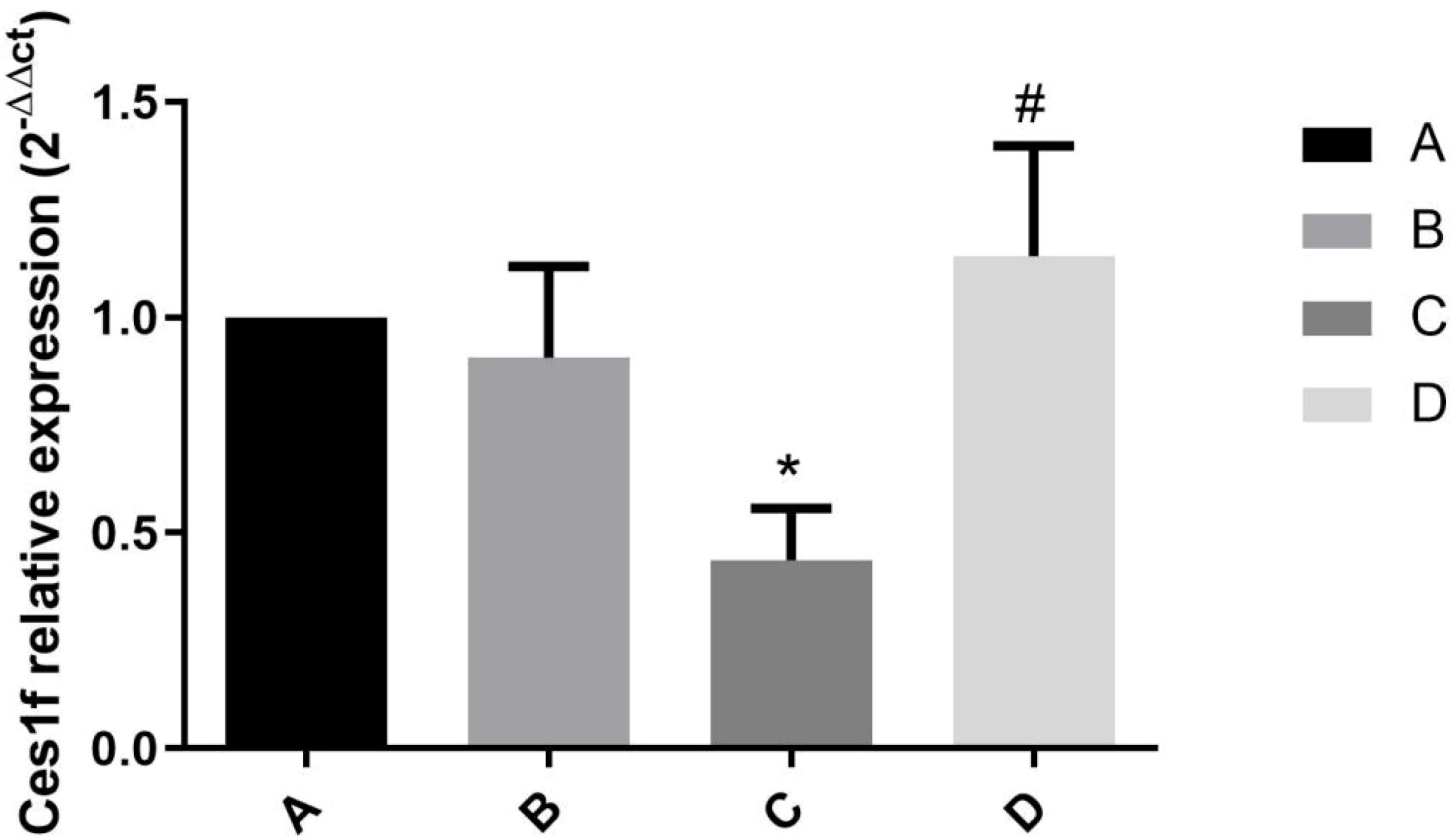
Expression of Ces1f mRNA in liver tissue of mice treated with LPS/D-GalN at 6h. **(A)** Urantide(-)LPS/D-GalN(-); **(B)** Urantide(+)LPS/D-GalN(-);**(C)** Urantide(-)LPS/D-GalN(+); **(D)**Urantide(+)LPS/D-GalN(+);*P<0.01 vs group A; #*P*<0.01 vs group C;The vertical axis represents the relative expression level of Ces1f 2^-△△ct^.

The relative expression of hepatic *Ces1f* mRNA exhibited significant variations across experimental groups (**Figure 4**), with values of 1.00 (Group A), 0.91 ± 0.31 (Group B), 0.44 ± 0.12 (Group C), and 1.14 ± 0.40 (Group D). Key findings include: (1) Group C showed a 56% reduction in *Ces1f* expression compared to Group A (*P* < 0.01), demonstrating LPS/D-GalN’s potent suppressive effect; (2) No significant difference existed between control Groups A and B (*P* = 0.861), confirming urantide’s neutral effect under physiological conditions; and (3) Group D exhibited a 2.6-fold higher expression than Group C (*P* < 0.01), with levels similar to Group A (*P* = 0.372), indicating urantide’s capacity to completely rescue *Ces1f* suppression during ALF. These results demonstrate that UII/UT system activation mediates *Ces1f* downregulation in ALF, while its pharmacological inhibition restores normal expression patterns, suggesting *Ces1f* as both a biomarker and potential therapeutic target in acute liver injury.

### 3.5 FISH Assay for the localization and expression levels of *Ces1f* mRNA in the liver

**Figure 5:**
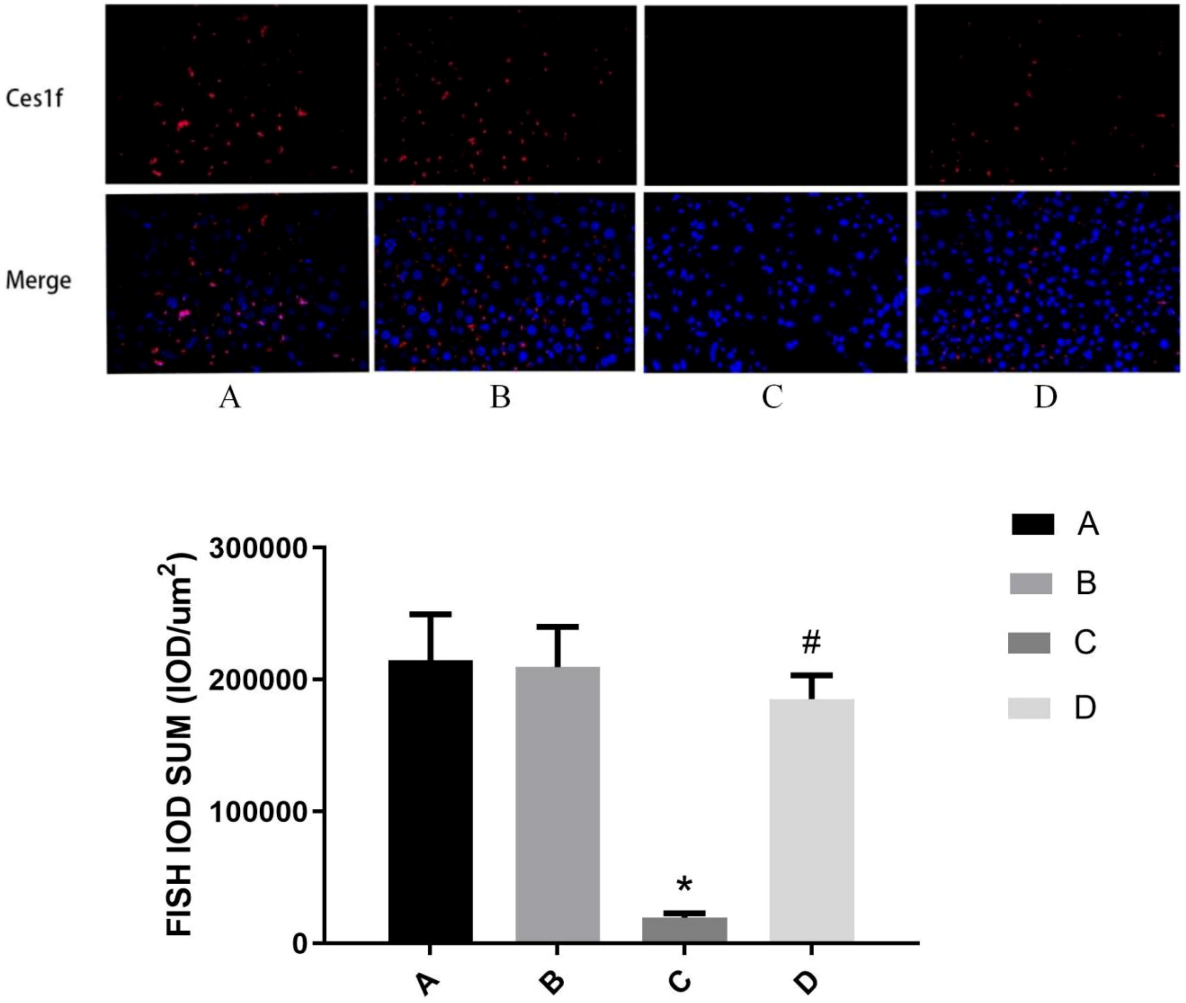
FISH Assay for the Localization and Expression Levels of *Ces1f* mRNA in the Liver. **(A)** Urantide(-)LPS/D-GalN(-); **(B)** Urantide(+),LPS/D-GalN(-); **(C)** Urantide(-),LPS/D-GalN(+); **(D)** Urantide(+),LPS/D-GalN(+);*P < 0.01 *vs* group A; #*P* < 0.01 *vs* group C.

Fluorescence *in situ* hybridization (FISH) analysis demonstrated that *Ces1f* mRNA was predominantly localized in the cytoplasmic compartment with negligible nuclear staining across all experimental groups. Quantitative fluorescence intensity revealed striking differences in expression levels among groups: Group A (214,708.99 ± 34,817.92), Group B (209,717.48 ± 30,330.57), Group C (19,736.74 ± 3,059.74) and Group D (185,306.75 ± 18,052.25). Statistical analysis showed Group C exhibited significantly lower expression than Groups A, B and D (all *P* < 0.01), while Group D demonstrated a remarkable 9.4-fold increase versus Group C (*P* < 0.01), with no difference observed between Groups A and B (*P* = 0.755). These results clearly indicate that: (1) ALF induces profound suppression of cytoplasmic *Ces1f* expression; (2) Urantide pretreatment effectively restores *Ces1f* to near-physiological levels; and (3) The UII/UT system specifically modulates *Ces1f* expression under pathological conditions without affecting basal levels, suggesting its role as a stress-responsive regulatory mechanism in hepatocytes (**Figure 5**). The subcellular localization pattern further supports Ces1f’s functional role in cytoplasmic metabolic processes that are disrupted during acute liver injury.

### 3.6 Expression of hepatic UII and Ces1f proteins in mice challenged by LPS/D-GalN via urantide pretreatment

**Figure 6:**
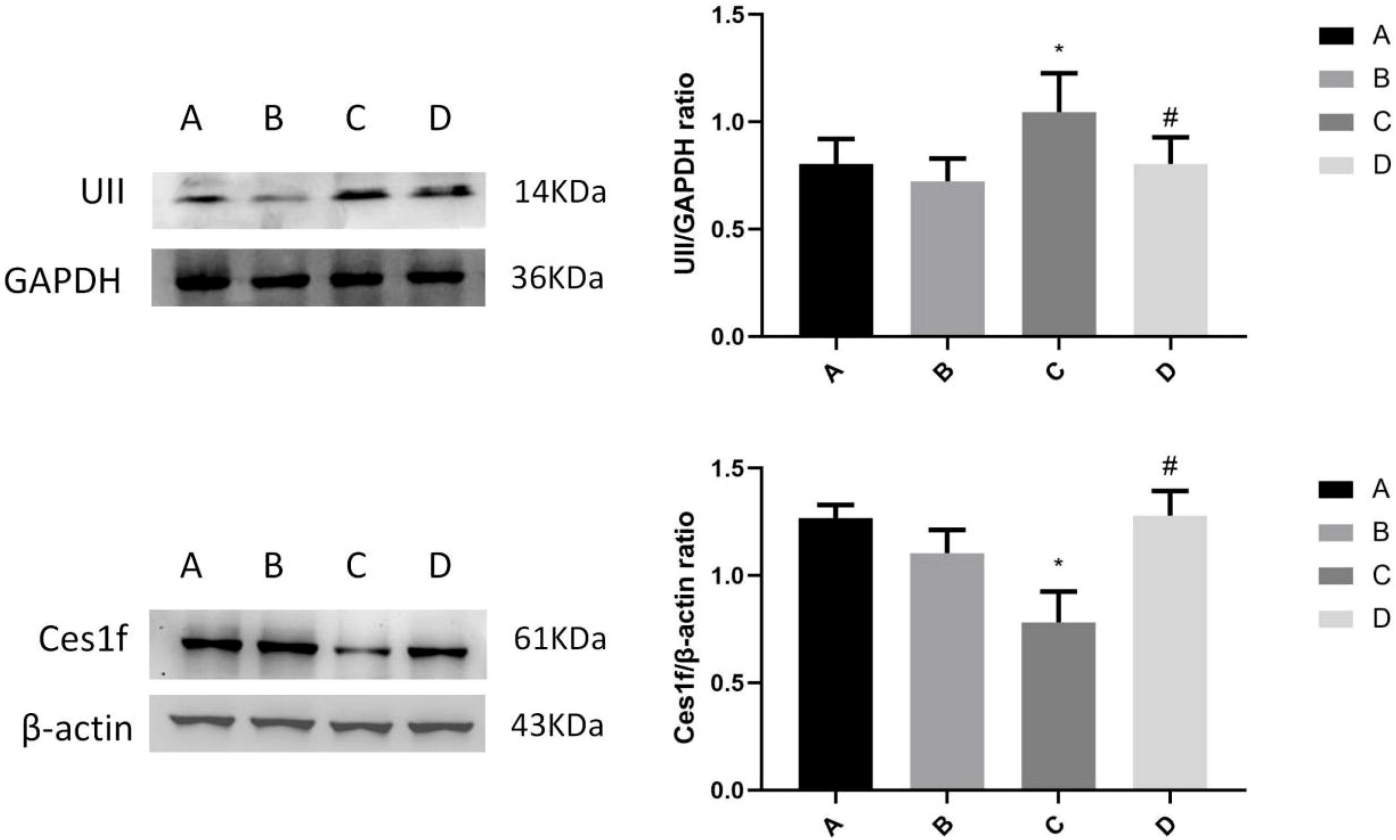
Western blotting detection of UII and Ces1f protein expression in liver tissue. **(A)** Urantide(-),LPS/D-GalN(-); **(B)** Urantide(+),LPS/D-GalN(-); **(C)** Urantide(-),LPS/D-GalN(+); **(D)** Urantide(+),LPS/D-GalN(+);*P < 0.05 *vs* group A; #*P* < 0.05 *vs* group C.

Western blot analysis revealed significant alterations of hepatic UII and Ces1f protein expression across experimental groups (**Figure 6**). Statistical analysis shows the levels of UII protein in mouse liver among groups:Group A (0.8043 ± 0.1161), Group B (0.7231 ± 0.1061), Group C (1.046 ± 0.1817), and Group D (0.8043 ± 0.1233). The UII protein expression in Group C showed a marked increase compared to Group A (normal control) (*P* < 0.05), while urantide pretreatment in Group D significantly attenuated this upregulation relative to Group C (*P* < 0.05). Notably, no difference in UII expression was observed between Groups A and B (*P* = 0.7276). Statistical analysis shows the levels of Ces1f protein in mouse liver among groups:Group A (1.268 ± 0.1730), Group B (1.104 ± 0.34446), Group C (0.7813 ± 0.3829), and Group D (1.279 ± 0.2818). Ces1f protein expression demonstrated an inverse pattern, with Group C exhibiting significant downregulation versus Group A (*P* < 0.05), and Group D showing restored expression levels compared to Group C (*P* < 0.05). The similar Ces1f expression between Groups A and B (*P* = 0.6784) confirmed that urantide administration does not affect basal protein levels in normal hepatocytes. These protein-level findings corroborate our mRNA expression data, demonstrating that: (1) ALF induces coordinated upregulation of UII and downregulation of Ces1f; (2) Urantide effectively normalizes these pathological alterations; and (3) The UII/UT signal blocker specifically modulates Ces1f expression during inflammatory injury without influencing physiological homeostasis, highlighting its potential as a therapeutic target for acute liver failure.

### 3.7 The levels of serum ALT and AST in mice challenged by LPS/D-GalN via urantide pretreatment

**Figure 7:**
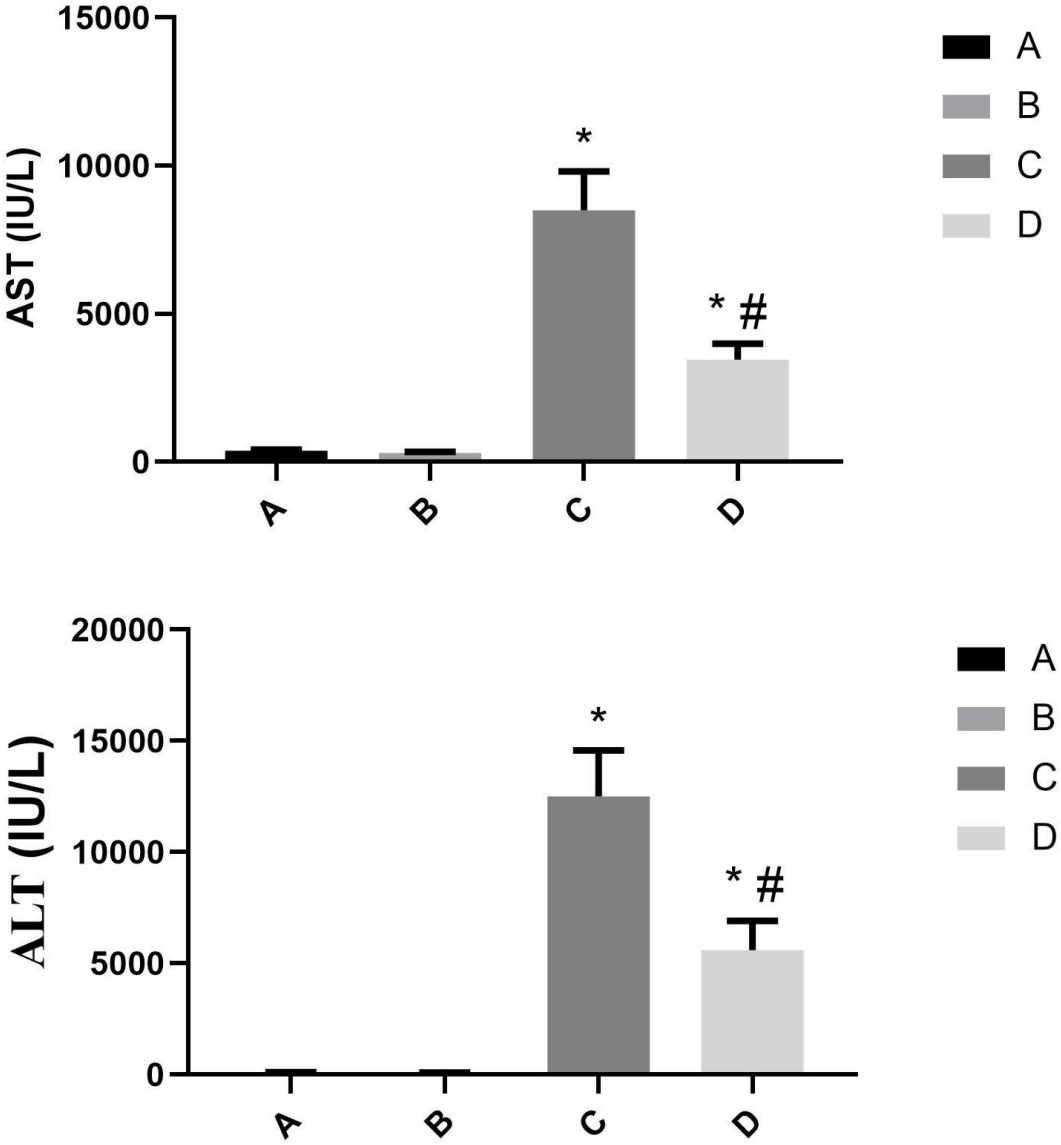
The levels of serum ALT and AST in LPS/D-GalN-Induced ALF Mice after urantide. *P < 0.01 *vs* group A; #*P* < 0.01 *vs* group C.

Serum biochemical analysis revealed marked differences in hepatic injury markers across experimental groups. The ALT levels were 94.63 ± 10.08 U/L (Group A), 85.13 ± 9.72 U/L (Group B), 12510.75 ± 2064.49 U/L (Group C), and 5593 ± 1316.53 U/L (Group D), while corresponding AST levels measured 380 ± 39.70 U/L, 309 ± 32.90 U/L, 8495 ± 1308.42 U/L, and 3455.25 ± 535.98 U/L, respectively. Statistical comparisons demonstrated that both LPS/D-GalN-challenged groups (C and D) exhibited significantly elevated ALT and AST levels compared to control groups (A and B) (*P* < 0.01). Notably, urantide pretreatment in Group D resulted in substantially attenuated transaminase elevations relative to untreated ALF mice (Group C), with reductions of 55.3% for ALT and 59.3% for AST (both *P* < 0.01) (**Figure 7**). These findings demonstrate that: (1) LPS/D-GalN successfully induced severe hepatocellular injury, and (2) UII/UT system blockade significantly ameliorates this damage, suggesting the therapeutic potential of urantide in acute liver failure.

### 3.8 Hepatic Histopathological changes in mice challenged by LPS/D-GalN via urantide pretreatment

**Figure 8:**
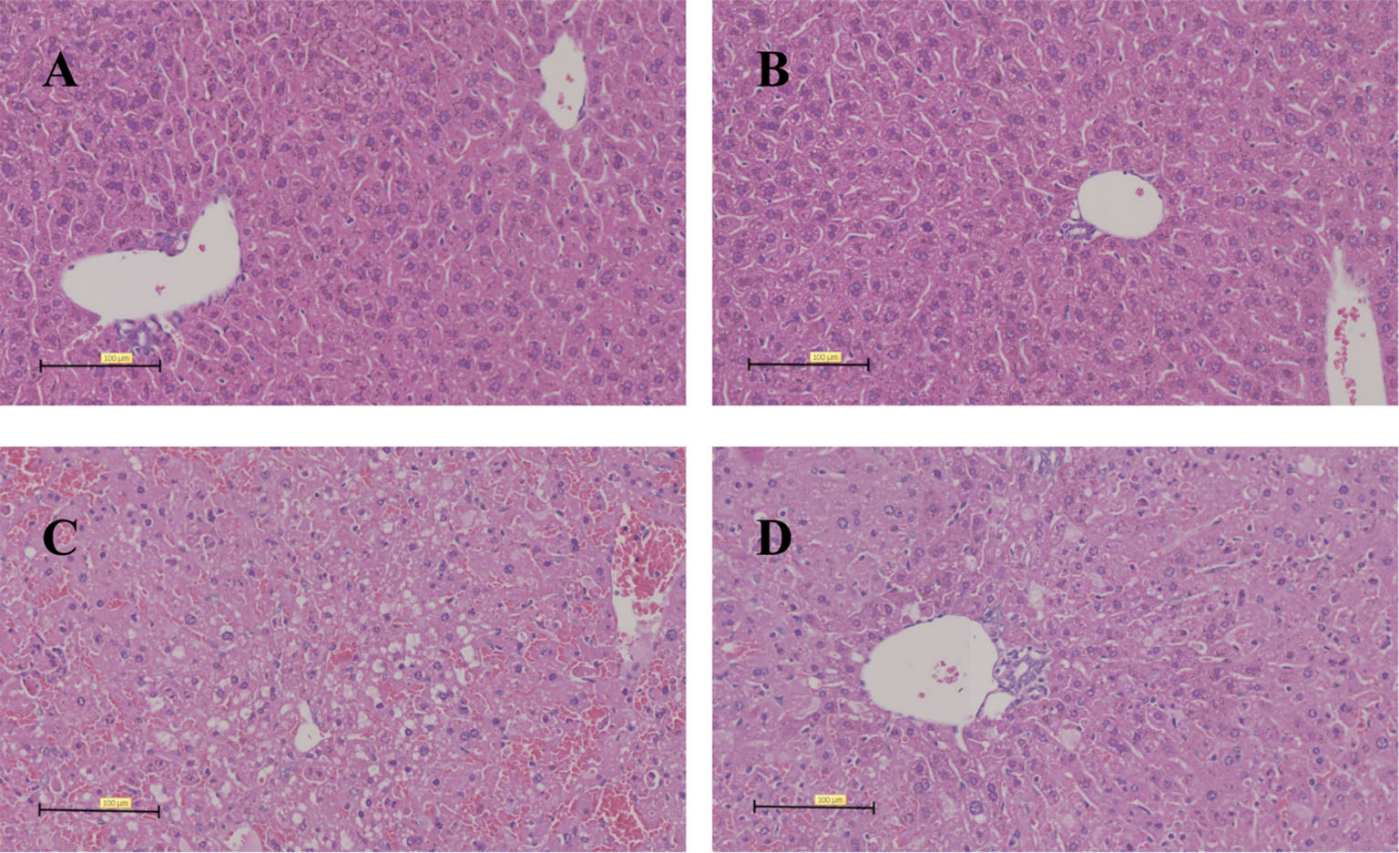
Histopathological changes in liver tissue of mice treated with LPS/D-GalN for 6 hours. **(A)** Urantide(-),LPS/D-GalN(-); **(B)** Urantide(+),LPS/D-GalN(-); **(C)** Urantide(-),LPS/D-GalN(+); **(D)** Urantide(+),LPS/D-GalN(+); (HE staining, x200).Scale bar=100 μm.

Histopathological examination demonstrated distinct morphological changes across experimental groups (**Figure 8**). Control groups (A and B) maintained normal hepatic architecture, with intact lobular structure and morphologically normal hepatocytes. In contrast, Group C (LPS/D-GalN-only) exhibited severe parenchymal destruction characterized by: (1) disrupted lobular architecture, (2) extensive necroinflammatory foci, (3) marked hepatocellular necrosis, (4) dense inflammatory infiltrates, and (5) pronounced sinusoidal congestion. Notably, urantide-pretreated Group D showed significant histological improvement compared to Group C, with attenuated sinusoidal congestion and reduced hepatocellular necrosis, indicating partial protection against LPS/D-GalN-induced injury through UII/UT system blockade. These findings correlate with the observed biochemical improvements and suggest that urantide modulates both inflammatory and cytotoxic components of ALF pathogenesis.

## 4 Discussion

UII, a neurohormone-like peptide primarily localized in KCs within the liver, has been identified as a key mediator in inflammatory diseases (Sun and Liu, 2019). Its biological effects are mediated through the UII/UT system, formed by binding to its specific receptor UT (Segain et al., 2007; Tomiyama et al., 2015). The uncontrolled release of proinflammatory cytokines from KCs leads to massive hepatocyte necrosis, constituting the primary pathophysiological mechanism of ALF (Maiwall et al., 2024).

Prior studies revealed that in ALF, both UII/UT levels and inflammatory cytokines (such as IL-6 and TNF-α) are significantly elevated (Liang et al., 2013; Leifeld et al., 2010). Mechanistically, UII/UT upregulation activates TLR4/p38-mitogen-activated protein kinase(p38-MAPK) and nuclear factor κB(NF-κB) signaling pathways, inducing excessive secretion of inflammatory mediators like IL-6 and TNF-α (Liu et al., 2015). Our studies further showed that serum UII secretion is an earlier event than TNF-α in ALF (Liu et al., 2015). It suggests that the UII/UT system induce hepatic injury by initiating the inflammatory cascade in ALF. Thus, the UII/UT system serves as a key trigger in LPS/D-GalN-induced ALF development. Previous reportor demonstrated that urantide protected against animals’ death and alleviated hepatic inflammatory injury by blocking UII/UT signaling in LPS/D-GalN-challenged ALF mice (Liang et al., 2013). In this experiment, hepatic inflammatory remission was again observed, including the decrease of serum ALT/AST and the improvement of hepatic tissue injury due to urantide pretreatment in ALF mice.

It is demonstred that during ALF, p38 MAPK/c-jun n-terminal kinase(JNK) remains persistently activated (Guan et al., 2025), and overactivated p38 MAPK and JNK phosphorylate peroxisome proliferator-activated receptor alpha (PPARα), thereby suppressing the transcriptional activity of PPARα bound to the Ces1f gene promoter region (Fang and Judd, 2018; Zhang et al., 2024; Wen et al., 2019). Furthermore, we found that hepatic Ces1f expression is significantly downregulated in ALF (Zhao et al., 2024). Ces1f, as a member of the carboxylesterase family (Lian et al., 2016), is known to mediate xenobiotic detoxification (Quiroga et al., 2012; Yang et al., 2009) and ester metabolism (Hosokawa et al., 2008; Yang et al., 2007). In previous experimental, we further observed that Ces1f was mainly expressed in KCs, and hepatic injury exacerbated upon knocking down KC *Ces1f* gene in LPS/D-GalN-attacked mice (Zhao et al., 2024). However, it is unclear whether there is an association between the UII/UT signal and Ces1f expression in ALF.

In order to learn about the relationship between the above two, we analysed the time-dependent expression of hepatic *UII* and *Ces1f* genes in LPS/D-GalN-induced ALF in this experiment. We found that after LPS/D-GalN attack, hepatic *UII* mRNA rose rapidly, reached the peak within 6 h, and returned to normal levels at 12 h; while the *Ces1f* declined progressively from 6 h, but began to rise at 12 h. Our previous study indicated that 6 h is an important time point of hepatic injury, at which serum ALT/AST began to rise and hepatocellular death, inflammatory infiltration and hemorrhage appeared after LPS/D-GalN attacked (Liu et al., 2008). Therefore, the marked hepatic damage may originate from both UII/UT activation and Ces1f down-regulation. In addition, it seems that it can be deduced to a regulating effect of UII on Ces1f because hepatic UII expression is a significant earlier event than Ces1f in ALF.

To further know whether hepatic UII/UT signal has an influence on Ces1f, we assayed hepatic Ces1f expression at 6 h after LPS/D-GalN challenge using urantide pretreatment. In this experimental, we observed that urantide pretreatment reversed hepatic UII upregulation and Ces1f downregulation induced by LPS/D-GalN challenge in ALF mice. It suggests that UII/UT system can regulate hepatic Ces1f in ALF.

Using our established LPS/D-GalN-induced ALF model (Sanghani et al., 2009; Liu et al., 2013), we found that: 1. During the acute liver failure (ALF) phase, the time-dependent expression levels of *UII* and *Ces1f* mRNA exhibited opposite trends: *UII* mRNA began to increase 2 hours after LPS/D-GalN stimulation, peaked at 6 hours, remained elevated at 8 hours, and decreased to near-normal levels by 12 hours; conversely, *Ces1f* mRNA showed a significant decrease starting at 6 hours post-stimulation, reached its lowest point at 10 hours, and began to rise again by 12 hours. 2. Compared to controls, 6 hours after LPS/D-GalN treatment, the ALF model group exhibited upregulation of *UII* mRNA and protein levels in the liver, while *Ces1f* mRNA and protein levels in the liver were downregulated. 3. Following urantide administration, compared to the model group, the pretreatment ALF group showed downregulation of *UII* mRNA and protein levels in the liver, while *Ces1f* mRNA and protein levels in the liver were upregulated. This suggests that the UII/UT signaling system may exacerbate ALF by suppressing the expression of hepatic metabolic enzymes such as Ces1f. However, several questions remain unresolved: the mechanism by which UII/UT influences Ces1f remains unclear and requires investigation using primary hepatocytes. Future studies will further explore the downstream signaling pathways of UII/UT and its regulatory mechanisms on the transcription of hepatic metabolic enzymes.

## Data Availability

The datasets presented in this study can be found in online repositories. The names of the repository/repositories and accession number(s) can be found in the article/**Supplementary Material**.

## References

[B1] Antoniades C. G. Khamri W. Abeles R. D. Taams L. S. Triantafyllou E. Possamai L. A. . (2014). Secretory leukocyte protease inhibitor: a pivotal mediator of anti-inflammatory responses in acetaminophen-induced acute liver failure. Hepatology. 59, 1564–1576. doi: 10.1002/hep.26933, PMID: 24282114

[B2] Becares N. Gage M. C. Voisin M. Shrestha E. Martin-Gutierrez L. Liang N. . (2019). Impaired LXRα Phosphorylation attenuates progression of fatty liver disease. Cell Rep. 26, 984–995.e6. doi: 10.1016/j.celrep.2018.12.094, PMID: 30673619 PMC6344342

[B3] Collins J. M. Lu R. Wang X. Zhu H. J. Wang D. (2022). Transcriptional regulation of carboxylesterase 1 in human liver: role of the nuclear receptor subfamily 1 group H member 3 and its splice isoforms. Drug Metab. Dispos. 50, 43–48. doi: 10.1124/dmd.121.000649, PMID: 34697082 PMC8969197

[B4] Crow J. A. Herring K. L. Xie S. Borazjani A. Potter P. M. Ross M. K. (2010). Inhibition of carboxylesterase activity of THP1 monocytes/macrophages and recombinant human carboxylesterase 1 by oxysterols and fatty acids. Biochim. Biophys. Acta 1801, 31–41. doi: 10.1016/j.bbalip.2009.09.002, PMID: 19761868 PMC2787731

[B5] Fang H. Judd R. L. (2018). Adiponectin regulation and function. Compr. Physiol. 8, 1031–1063. doi: 10.1002/cphy.c170046, PMID: 29978896

[B6] Guan J. Wu F. Wu S. Ren Y. Wang J. Zhu H. (2025). FTY720 alleviates D-GalN/LPS-induced acute liver failure by regulating the JNK/MAPK pathway. Int. Immunopharmacol. 157, 114726. doi: 10.1016/j.intimp.2025.114726, PMID: 40311319

[B7] He Y. Zhong H. Yang X. Shi Q. Q. Xu G. R. Liu L. M. (2019). An effect of urantide on hepatic p120-catenin expression in acute hepatic failure mice. J. Nanjing Med. University(Natural Sciences)(in Chinese) 39, 495–498. doi: 10.7655/NYDXBNS20190406

[B8] Hosokawa M. Furihata T. Yaginuma Y. Yamamoto N. Watanabe N. Tsukada E. . (2008). Structural organization and characterization of the regulatory element of the human carboxylesterase (CES1A1 and CES1A2) genes. Drug Metab. Pharmacokinet. 23, 73–84. doi: 10.2133/dmpk.23.73, PMID: 18305377

[B9] Leifeld L. Clemens C. Heller J. Trebicka J. Sauerbruch T. Spengler U. (2010). Expression of urotensin II and its receptor in human liver cirrhosis and fulminant hepatic failure. Digestive Dis. Sci. 55, 1458–1464. doi: 10.1007/s10620-009-0875-4, PMID: 19582578

[B10] Li X. Zhi Y. Li J. Lei X. Ju Y. Zhang Y. . (2023). Single-cell RNA sequencing to reveal non-parenchymal cell heterogeneity and immune network of acetaminophen-induced liver injury in mice. Arch. Toxic 97, 1979–1995. doi: 10.1007/s00204-023-03513-4, PMID: 37202523

[B11] Lian J. Wei E. Groenendyk J. Das S. K. Hermansson M. Li L. . (2016). Ces3/TGH deficiency attenuates steatohepatitis. Sci. Rep. 6, 25747. doi: 10.1038/srep25747, PMID: 27181051 PMC4867576

[B12] Liang D. Y. Liu L. M. Ye C. G. Zhao L. Yu F. P. Gao D. Y. . (2013). Inhibition of UII/UTR system relieves acute inflammation of liver through preventing activation of NF-κB pathway in ALF mice. PloS One 8, e64895. doi: 10.1371/journal.pone.0064895, PMID: 23755157 PMC3670940

[B13] Liu Y. Li J. Zhu H. J. (2024). Regulation of carboxylesterases and its impact on pharmacokinetics and pharmacodynamics: an up-to-date review. Expert Opin. Drug Metab. Toxicol. 20, 377–397. doi: 10.1080/17425255.2024.2348491, PMID: 38706437 PMC11151177

[B14] Liu L. M. Liang D. Y. Ye C. G. Tu W. J. Zhu T. (2015). The UII/UT system mediates upregulation of proinflammatory cytokines through p38 MAPK and NF-κB pathways in LPS-stimulated Kupffer cells. PloS One 10, e0121383. doi: 10.1371/journal.pone.0121383, PMID: 25803040 PMC4372515

[B15] Liu L. M. Ye C. G. Liang D. Y. Yu F. P. Zhao L. Sun S. L. . (2013). Effects of urantideas a UT receptor antagonist on the signal molecules of NF-κB pathway in mice with acute liver failure. Chin. Hepatol. 18, 88–91. doi: 10.3969/j.issn.1008-1704.2013.02.006

[B16] Liu L. M. Zhang J. X. Luo J. Guo H. X. Deng H. Chen J. Y. . (2008). A role of cell apoptosis in lipopolysaccharide (LPS)-induced nonlethal liver injury in D-galactosamine (D-GalN)-sensitized rats. Dig Dis. Sci. 53, 1316–1324. doi: 10.1007/s10620-007-9994-y, PMID: 17934810

[B17] Maiwall R. Kulkarni A. V. Arab J. P. Piano S. (2024). Acute liver failure. Lancet (London England) 404, 789–802. doi: 10.1016/S0140-6736(24)00693-7, PMID: 39098320

[B18] Mangum L. C. Hou X. Borazjani A. Lee J. H. Ross M. K. Crow J. A. (2018). Silencing carboxylesterase 1 in human THP-1 macrophages perturbs genes regulated by PPARγ/RXR and RAR/RXR: down-regulation of CYP27A1-LXRα signaling. Biochem. J. 475, 621–642. doi: 10.1042/BCJ20180008, PMID: 29321244 PMC6159944

[B19] Parlar Y. E. Ayar S. N. Cagdas D. Balaban Y. H. (2023). Liver immunity, autoimmunity, and inborn errors of immunity. World J. Hepatol. 15, 52–67. doi: 10.4254/wjh.v15.i1.52, PMID: 36744162 PMC9896502

[B20] Quiroga A. D. Li L. Trötzmüller M. Nelson R. Proctor S. D. Köfeler H. . (2012). Deficiency of carboxylesterase 1/esterase-x results in obesity, hepatic steatosis, and hyperlipidemia. Hepatol. (Baltimore Md.) 56, 2188–2198. doi: 10.1002/hep.25961, PMID: 22806626

[B21] Sanghani S. P. Sanghani P. C. Schiel M. A. Bosron W. F. (2009). Human carboxylesterases: an update on CES1, CES2 and CES3. Protein Pept. Lett. 16, 1207–1214. doi: 10.2174/092986609789071324, PMID: 19508181

[B22] Satoh T. Hosokawa M. (1998). The mammalian carboxylesterases: from molecules to functions. Annu. Rev. Pharmacol. Toxicol. 38, 257–288. doi: 10.1146/annurev.pharmtox.38.1.257, PMID: 9597156

[B23] Satoh T. Taylor P. Bosron W. F. Sanghani S. P. Hosokawa M. La Du B. N. (2002). Current progress on esterases: from molecular structure to function. Drug Metab. disposition: Biol. fate chemicals 30, 488–493. doi: 10.1124/dmd.30.5.488, PMID: 11950776

[B24] Segain J. P. Rolli-Derkinderen M. Gervois N. Raingeard de la Blétière D. Loirand G. Pacaud P. (2007). Urotensin II is a new chemotactic factor for UT receptor-expressing monocytes. J. Immunol. (Baltimore Md.: 1950) 179, 901–909. doi: 10.4049/jimmunol.179.2.901, PMID: 17617581

[B25] Sun S. L. Liu L. M. (2019). Urotensin II: an inflammatory cytokine. J. Endocrinol. 240 (3), R107–R117. doi: 10.1530/JOE-18-0505, PMID: 30601760

[B26] Tomiyama S. Nakamachi T. Uchiyama M. Matsuda K. Konno N. (2015). Urotensin II upregulates migration and cytokine gene expression in leukocytes of the African clawed frog, Xenopus laevis. Gen. Comp. Endocrinol. 216, 54–63. doi: 10.1016/j.ygcen.2015.04.009, PMID: 25907658

[B27] Triantafyllou E. Pop O. T. Possamai L. A. Wilhelm A. Liaskou E. Singanayagam A. . (2018a). MerTK expressing hepatic macrophages promote the resolution of inflammation in acute liver failure. Gut. 67, 333–347. doi: 10.1136/gutjnl-2016-313615, PMID: 28450389 PMC5868289

[B28] Triantafyllou E. Woollard K. J. McPhail M. J. W. Antoniades C. G. Possamai L. A. (2018b). The role of monocytes and macrophages in acute and acute-on-chronic liver failure. Front. Immunol. 9. doi: 10.3389/fimmu.2018.02948, PMID: 30619308 PMC6302023

[B29] Wen X. Baker A. A. Klaassen C. D. Corton J. C. Richardson J. R. Aleksunes L. M. (2019). Hepatic carboxylesterases are differentially regulated in PPARα-null mice treated with perfluorooctanoic acid. Toxicology. 416, 15–22. doi: 10.1016/j.tox.2019.01.014, PMID: 30685356 PMC6397673

[B30] Wu Z. Han M. Chen T. Yan W. Ning Q. (2010). Acute liver failure: mechanisms of immune-mediated liver injury. Liver Int. 30 (6), 782–794. doi: 10.1111/j.1478-3231.2010.02262.x, PMID: 20492514

[B31] Yang D. Pearce R. E. Wang X. Gaedigk R. Wan Y. J. Yan B. (2009). Human carboxylesterases HCE1 and HCE2: ontogenic expression, inter-individual variability and differential hydrolysis of oseltamivir, aspirin, deltamethrin and permethrin. Biochem. Pharmacol. 77, 238–247. doi: 10.1016/j.bcp.2008.10.005, PMID: 18983829 PMC2671154

[B32] Yang J. Shi D. Yang D. Song X. Yan B. (2007). Interleukin-6 alters the cellular responsiveness to clopidogrel, irinotecan, and oseltamivir by suppressing the expression of carboxylesterases HCE1 and HCE2. Mol. Pharmacol. 72, 686–694. doi: 10.1124/mol.107.036889, PMID: 17537833

[B33] Yu Q. Q. Cheng D. X. Xu L. R. Li Y. K. Zheng X. Y. Liu Y. . (2020). Urotensin II and urantide exert opposite effects on the cellular components of atherosclerotic plaque in hypercholesterolemic rabbits. Acta Pharmacol. Sin. 41, 546–553. doi: 10.1038/s41401-019-0315-8, PMID: 31685976 PMC7468446

[B34] Zhang Y. He X. Gu L. Li S. Tang J. Ma R. . (2024). Mefunidone ameliorates acute liver failure in mice by inhibiting MKK4-JNK pathway. Biochem. Pharmacol. 225, 116267. doi: 10.1016/j.bcp.2024.116267, PMID: 38723721

[B35] Zhao S. Yang X. He Y. Yu Q. Liu L. M. (2024). Kupffer-cell-targeted carboxylesterase 1f knockdown deteriorates lipopolysaccharide/D-galactosamine-induced acute liver failure through regulating cellular polarization in mice. Can. J. Gastroenterol. Hepatol. 2024, 6410484. doi: 10.1155/cjgh/6410484, PMID: 39734640 PMC11681982

