# Supplementary figures and images for: Urantide alleviates lipopolysaccharide/D-galactosamine-induced acute liver failure through upregulating carboxylesterase1f in mice
Source: Front Cell Infect Microbiol. 2025 Dec 17;15:1653725. doi: 10.3389/fcimb.2025.1653725 (PMC12753892; doi:10.3389/fcimb.2025.1653725)

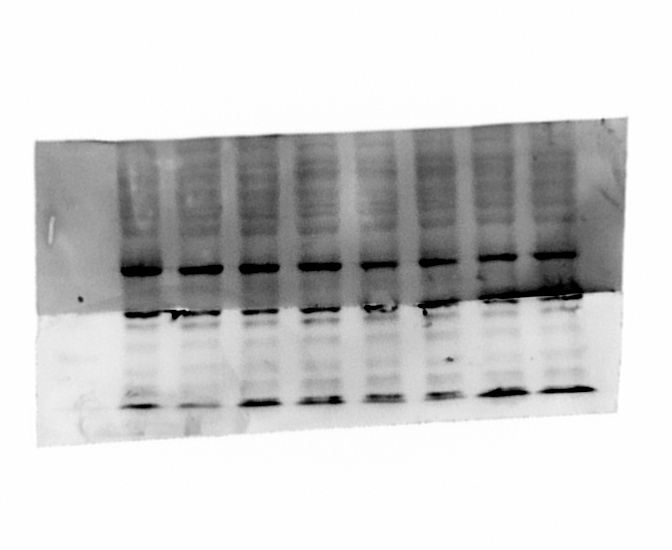

Supplement: Supplementary file 1 [file Image1.tif]

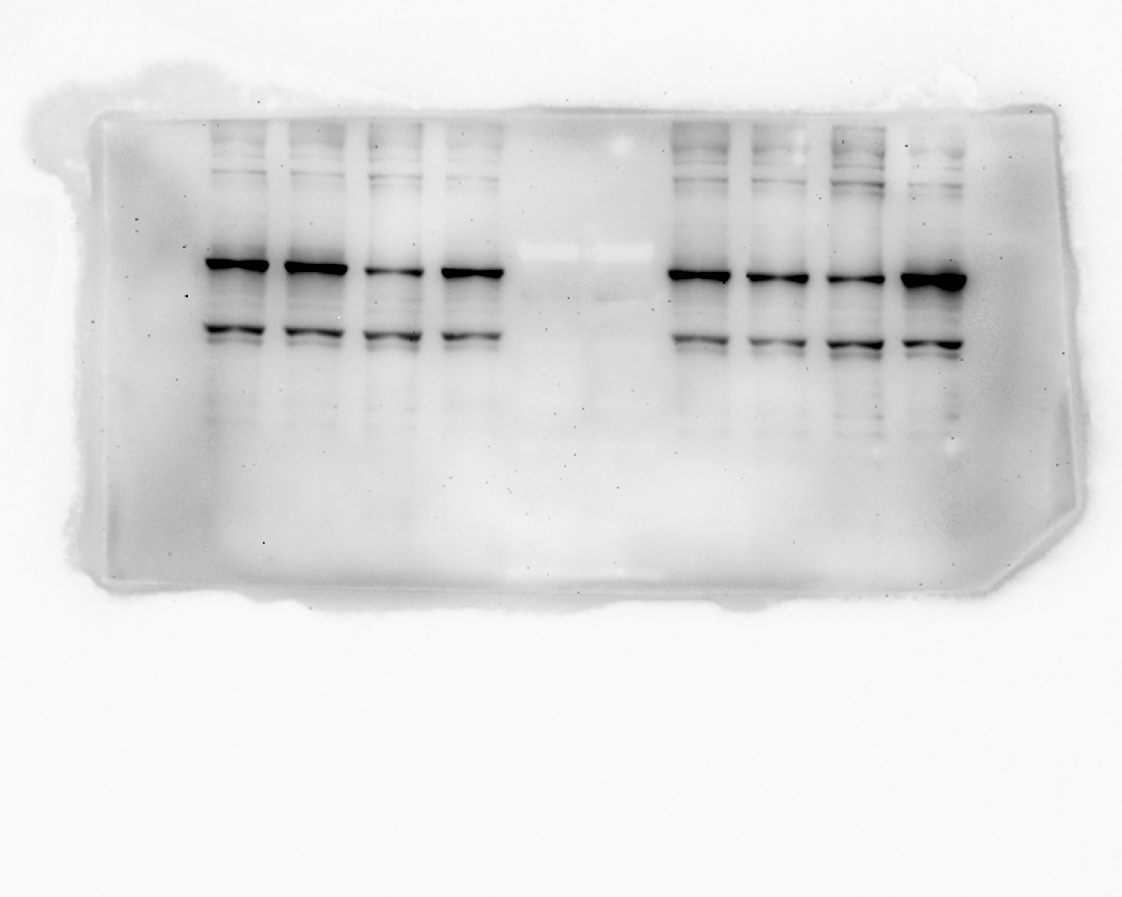

Supplement: Supplementary file 2 [file Image2.tif]

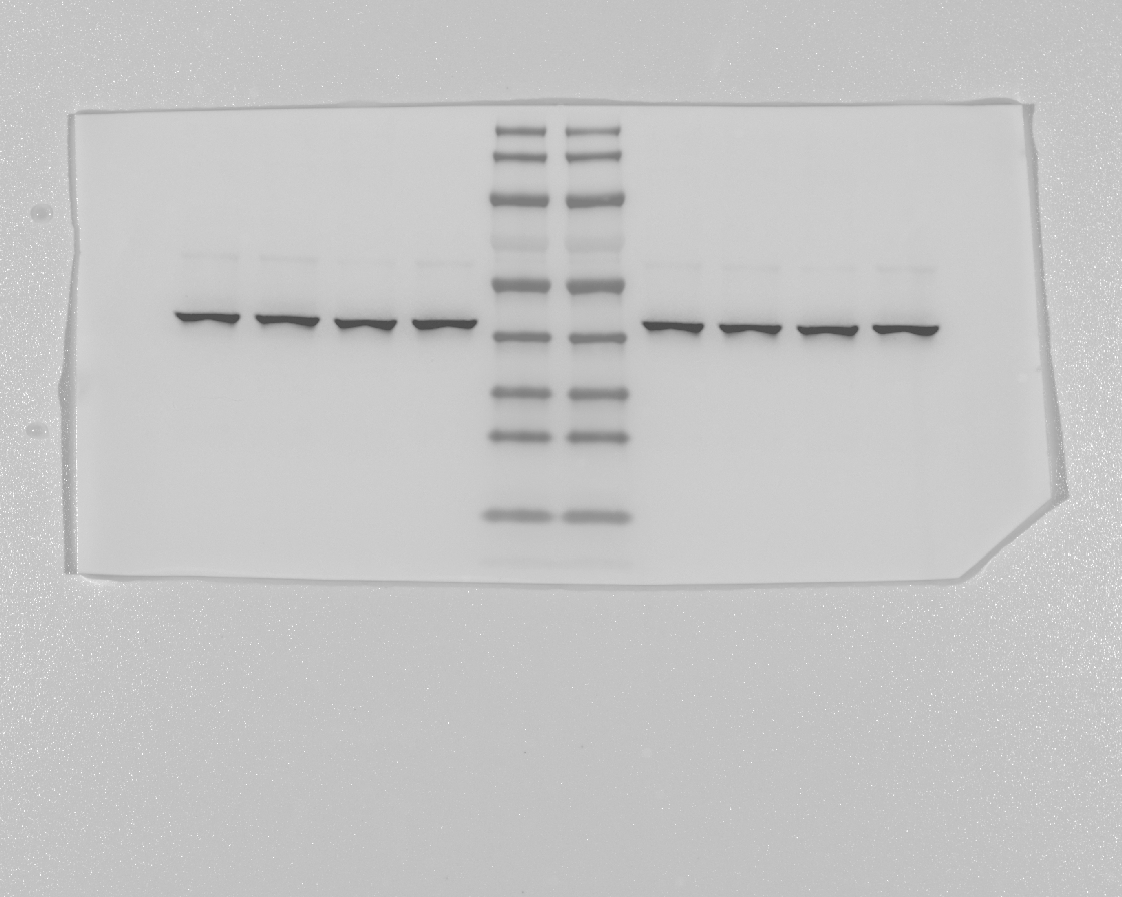

Supplement: Supplementary file 3 [file Image3.jpeg]
